# The Potential Effect of Aberrant Testosterone Levels on Common Diseases: A Mendelian Randomization Study

**DOI:** 10.3390/genes11070721

**Published:** 2020-06-29

**Authors:** Ali Alamdar Shah Syed, Lin He, Yongyong Shi

**Affiliations:** 1 Bio-X Institutes, Key Laboratory for the Genetics of Developmental and Neuropsychiatric Disorders (Ministry of Education), Shanghai Jiao Tong University, 1954 Huashan Road, Shanghai 200030, China; helin@sjtu.edu.cn (L.H.); shiyongyong@gmail.com (Y.S.); 2 Shanghai Center for Women and Children’s Health, 339 Luding Road, Shanghai 200062, China

**Keywords:** Mendelian randomization (MR), testosterone, schizophrenia (SCZ), Alzheimer’s disorder (AD), type II diabetes (T2D), gout

## Abstract

Testosterone has historically been linked to sexual dysfunction; however, it has recently been shown to affect other physical and mental attributes. We attempted to determine whether changes in serum testosterone could play a role in chronic or degenerative diseases. We used two separate genetic instruments comprising of variants from *JMJD1C* and *SHBG* regions and conducted a two-sample Mendelian randomization for type II diabetes (T2D), gout, rheumatoid arthritis (RA), schizophrenia, bipolar disorder, Alzheimer’s disease and depression. For the *JMJD1C* locus, one unit increase in log transformed testosterone was significantly associated with RA (OR = 1.69, *p* = 0.02), gout (OR = 0.469, *p* = 0.001) and T2D (OR = 0.769, *p* = 0.048). Similarly, one unit increase in log transformed testosterone using variants from the *SHBG* locus was associated with depression (OR = 1.02, *p* = 0.001), RA (OR = 1.32, *p* < 0.001) and T2D (OR = 0.88, *p* = 0.003). Our results show that low levels of serum testosterone levels may cause gout and T2D, while higher than normal levels of testosterone may result in RA and depression. Our findings suggest that fluctuations in testosterone levels may have severe consequences that warrant further investigation.

## 1. Introduction

Testosterone is a universally known hormone which is responsible for the development of primary, as well as secondary male characteristics, such as genitalia, beards and muscle mass [1]. Historically, the role of testosterone was thought to be limited to the development of male characteristics and sexual function; however, recently it has come to light that the role of testosterone may be multifarious. Research has reported testosterone to be essential to male health, with some studies showing mortality rates to be as much as 40% higher in individuals with extremely low testosterone levels [2,3]. Studies conducted over the previous decades have discovered that testosterone has the potential to influence men both physically and psychologically, affecting the overall health of the male population.

Male hypogonadism is defined by The Endocrine Society as the inability to produce physiological levels of testosterone and is clinically defined as an early morning testosterone concentration of 250–300 ng/dL or below [4]. Hypogonadism can be present at any phase of life, from fetal development to old age; however, it is known that serum testosterone levels steadily decrease as men age [5,6] and is symptomized by decreased libido, erectile dysfunction, decreased muscle mass and bone mineral density, all of which were previously dismissed as a natural consequence of aging. Testosterone levels begin to naturally decrease between the ages of 20 and 30 in males, at a rate of about 1.3% per year [7], and by the age of 45, about 39% of men have serum testosterone levels below 300 ng/dL [8]. An investigation into testosterone tests undertaken by individuals in United Kingdom and the United States, from the years 2000 to 2011, shows that male hypogonadism is quite common, with an excess of 20% of tests displaying a low level of serum testosterone (< 300 mg/dL). This phenomenon is not only restricted to the elderly, as up to 76% of participants were between the ages of 40 and 64 [9].

Increasing awareness of hypogonadism and its association with common conditions such as type II diabetes mellitus, obesity and metabolic syndrome, ultimately gave rise to testosterone replacement therapy (TRT) in an attempt to reverse the effects of hypogonadism. A recent meta-analysis randomized control trial (RCT) reports that TRT can improve quality of life, libido, depression and erectile dysfunction [10]; further studies report the positive effects of TRT on obesity, lipid profile and cardiovascular risk factors [11]. The safety of testosterone supplement usage has been called into question, with increased incidents of cardiovascular events observed in patients undergoing therapy [12]. Two large-scale observational studies have since confirmed increased occurrence of cardiovascular events as a result of TRT [13,14], leading Health Canada and the US Food and Drug Administration (FDA) to place labels on TRT products in an attempt to warn the public of the potentially adverse cardiological effects of TRT.

Testosterone has also been shown to greatly influence male behavior, particularly aggressive behaviors which can termed as antisocial, while also stimulating generosity when it can serve to increase the male’s dominance [15,16]. Over the past few decades, testosterone has been consistently linked to cognitive decline, particularly in the elderly [17]. Results from several epidemiological studies have suggested that testosterone acts as a metabolic hormone in males, as there is a negative correlation between testosterone levels (both free as well as *SHBG* bound) and obesity across all age groups [18,19,20,21].

Mendelian randomization provides us with a technique that can uncover causal relationships without the extensive costs incurred by randomized control trials, and is becoming increasingly more viable as data from numerous large genome wide association studies (GWAS) investigating a diverse range of conditions over the past decade are now publicly available. Early Mendelian randomization studies involving testosterone were performed in small samples and were subsequently met with little success [22,23,24]; recent studies that shifted to the use of large-scale GWAS to source associations of variant with exposure and outcome have reported significant findings regarding testosterone levels and the risk of stroke, cardiovascular disease, polycystic ovary syndrome, as well as breast, endometrial and prostate cancer [25,26].

It is now obvious that the functions of testosterone extend well beyond the sexual domain, especially in men, affecting both physiological and mental aspects of life. Hypogonadism is widespread and inevitable; however, it is difficult to conduct studies to ascertain the full extent of testosterone influence, which has been hypothesized to play a role in a range of common metabolic, immune and psychiatric diseases [27,28,29,30,31,32,33].

In this study, we aim to determine the effect of testosterone on previously associated common diseases, namely, type II diabetes (T2D), rheumatoid arthritis (RA), gout, depression, bipolar disorder (BP), schizophrenia (SCZ) and Alzheimer’s disorder (AD) [27,28,29,30,31,32,33], by conducting a two-sample Mendelian randomization study in an effort to determine whether testosterone could potentially play a causative role in these disorders. To our knowledge, this is the first Mendelian randomization study looking into the relationship between testosterone and common diseases.

## 2. Materials and Methods

### 2.1. Genetic Instruments (Genetic Associations with Testosterone)

The genetic instruments for testosterone were created using data from the REDUCE study, where 3225 men of European descent between the ages of 50 and 75 years had consented to additional genetic testing [34]. The only variants significantly associated with serum testosterone at a genome-wide association level were present at 10q21, 17p13, Xp22, and were henceforth referred to as the *JMJD1C*, *SHBG* and *FAM9B* loci, respectively. Variants from the *FAM9B* locus were not included in our analysis since genetic variants in outcome data did not include the sex chromosomes. The *SHBG*, also known as sex hormone binding globulin, has a mechanistic link to all sex hormones and testosterone is no different, while the *JMJD1C* locus is associated with testosterone levels independent of *SHBG*.

Genetic instruments were created for both *JMJD1C* and *SHBG* loci separately, and a total of 661 and 325 variants were reported for the *JMJD1C* and *SHBG* loci, respectively. The genetic instrument for the *JMJD1C* locus was created based on a single genome-wide significant variant while the instrument for the *SHBG* locus was created using a step-wise selection procedure similar to the one used in a previous Mendelian randomization study [26]. In brief, for the *SHBG* locus, from the list of 325 variants, the strongest variant in terms of *p*-value was selected, and all other variants that were in linkage disequilibrium (defined as having an *r*^2^ value of > 0.40) were removed, after which the process was repeated for the next most significant variant, resulting in a set of variants with low pair-wise correlations. 

### 2.2. Genetic Association with Outcomes

Genetic association data for the selected outcomes were sourced from the GWAS catalogue. In this study, we investigated the association of the genetic instrument with various outcomes, namely, type 2 diabetes (T2D), gout, rheumatoid arthritis (RA), depression, schizophrenia, bipolar disorder and Alzheimer’s disorder. The genetic associations for T2D were extracted from a recently published meta-analysis of GWA studies that comprised approximately 16 million variants and included 62,892 cases along with 596,424 controls of European descent [35]. This meta-analysis was comprised of data from DIAGRAM, GERA and UK Biobank data sets.

The second outcome was derived from a study by Tin et al. [36], who performed a transancestry meta-analysis of serum urate levels (457,690 individuals) and made a subsequent prediction of gout in a separate cohort of 334,880 individuals from the UK Biobank. For RA, the genetic associations were extracted from a meta-analysis of GWA studies that evaluated approximately 10 million single nucleotide polymorphisms (SNPs), with total sample size >100,000 individuals (of European and Asian descent) comprising 29,880 and 73,758 cases and controls, respectively [37]. 

For depression, we used the genetic associations from the UK biobank [38], which has divided depression into three distinct phenotypes, mainly broad depression, probable major depressive disorder (MDD) and MDD as defined by the International Classification of Diseases (ICD). Here we selected genetic associations for broad depression as the outcome measure, as it most closely related to general depression experienced by most individuals. The UK Biobank depression GWAS was comprised of 322,580 participants. Genetic associations for schizophrenia were extracted from the GWAS published by the schizophrenia working group of the Psychiatric Genomics Consortium (PGC) [39], which is the largest GWAS performed on a European population today, comprising 36,989 cases and 113,075 controls. 

The genetic associations for bipolar disorder were extracted from a recent GWAS that was published in 2019, comprising 20,352 and 31,358 cases and controls, which was followed up with a separate group of an additional 9412 cases and 137,760 controls. A recently published meta-analysis of Alzheimer’s disorder studies comprising 67,614 cases was used to extract genetic associations with Alzheimer’s disorder. This meta-analysis combined data from the UK Biobank and International Genomics of Alzheimer’s Project (IGAP)

### 2.3. Statistical Analysis

Mendelian randomization is a technique that can be used to determine causal exposure–outcome relationships. Since alleles are randomly assigned at birth, genetic variants that are associated with an increase in exposure can be used to determine whether the said exposure could cause disease, while being completely randomized for confounders such as smoking or education, similar to a randomized control trial. 

The genetic associations and outcomes were harmonized before the Mendelian randomization analysis, which was performed separately from the two loci (*SHBG* and *JMJD1C*). The Mendelian randomization analysis was performed using the inverse variance (IVW) method: if the genetic instrument is comprised of a single variant then the resulting causal estimate is simply the ratio of association of variant with outcome and exposure. If the genetic instrument consists of more than one variant, then the causal estimate is calculated by pooling the ratio estimates using an inverse variance meta-analysis. 

Mendelian randomization analysis requires that genetic instruments utilized are associated with the outcome of interest only via the exposure, and is not associated with other exposures or confounders. In simple terms, Mendelian randomization requires that the genetic instrument used does not display pleiotropy; a test directional pleiotropy can be performed using the MR-Egger intercept estimate [40]. Two-sided *p*-values were reported for all analyses and significant casual estimates were defined as estimates with a *p*-value of < 0.05. Additional sensitivity analyses were performed using the weighted median method, which is capable of providing a consistent estimate even if up to 50% of the genetic instrument is invalid. All analyses were performed using the Mendelian randomization package in R.

## 3. Results

### 3.1. Genetic Instruments

After accounting for linkage disequilibrium, the genetic instrument for the *JMJD1C* locus was comprised of a single SNP (rs10822184), while the *SHBG* locus comprised of 20 variants (Table 1); however, a different set of variants was used depending on availability in the outcome data set. The exact composition of the genetic instruments and the pairwise correlations of the included variants can be found in the supplementary files (Figures S1–S3 and Tables S1–S3).

### 3.2. Mendelian Randomization of the JMJD1C Locus

**Figure 1:**
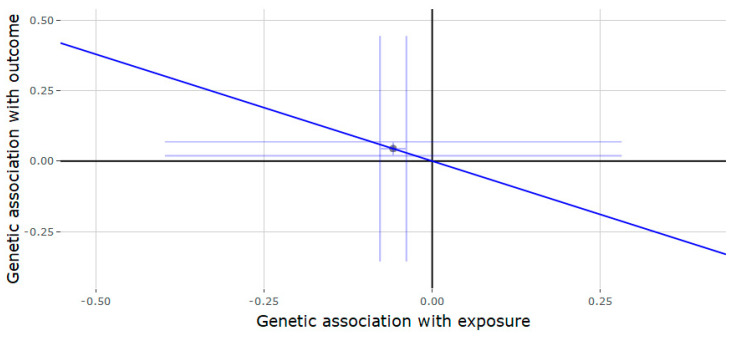
Genetic association of gout against genetic association of testosterone (*JMJD1C* locus). The slope (dark blue line) represents the causal association of testosterone with gout (generated via inverse variance weighted Mendelian randomization), while the point on the plot and the lights around it (light blue lines) represent the SNP and its confidence intervals.

**Figure 2:**
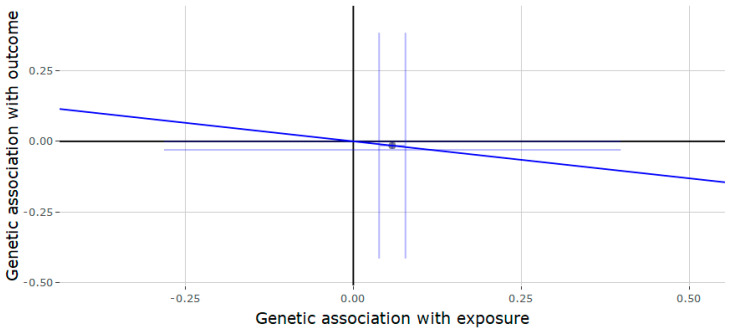
Genetic association of type II diabetes (T2D) against genetic association of testosterone (*JMJD1C* locus). The slope (dark blue line) represents the causal association of testosterone with T2D (generated via inverse variance weighted Mendelian randomization), while the point on the plot and the lights around it (light blue lines) represent the SNP and its confidence intervals.

**Figure 3:**
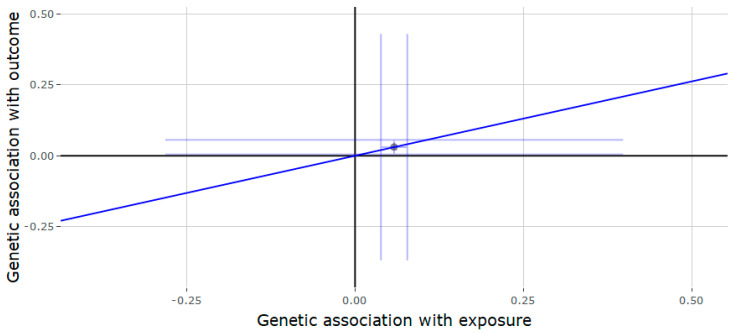
Genetic association of rheumatoid arthritis (RA) against genetic association of testosterone (*JMJD1C* locus). The slope (dark blue line) represents the causal association of testosterone with RA (generated via inverse variance weighted Mendelian randomization), while the point on the plot and the lights around it (light blue lines) represent the SNP and its confidence intervals.

The results of the Mendelian randomization or the *JMJD1C* locus (Table 2) showed that genetically predicted testosterone was negatively associated with gout (estimate = −0.757, 95% CI = −1.189, −0.324, *p* = 0.001) (Figure 1) and T2D (estimate = −0.262, 95% CI = −0.522, −0.002, *p* = 0.048) (Figure 2). Each increase in SD for genetically determined serum testosterone levels was positively associated with RA (estimate = 0.525, 95% CI = 0.083, 0.967, *p* = 0.020) (Figure 3). There was no association between genetically predicted testosterone and the following outcomes: Alzheimer disorder, bipolar disorder, schizophrenia and depression. Since the genetic instrument for the *JMJD1C* locus was comprised of only a single variant, it was not possible to conduct the sensitivity analyses (weighted median and MR-Egger methods). 

### 3.3. Mendelian Randomization of the SHBG Locus

**Figure 4:**
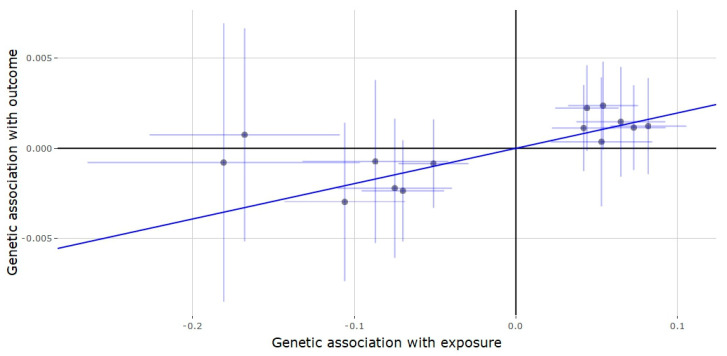
Genetic association of depression against genetic association of testosterone (*SHBG* locus). The slope (dark blue line) represents the causal association of testosterone with depression (generated via inverse variance weighted Mendelian randomization), while the points on the plot and the lights around them (light blue lines) represent the SNP and its confidence intervals.

**Figure 5:**
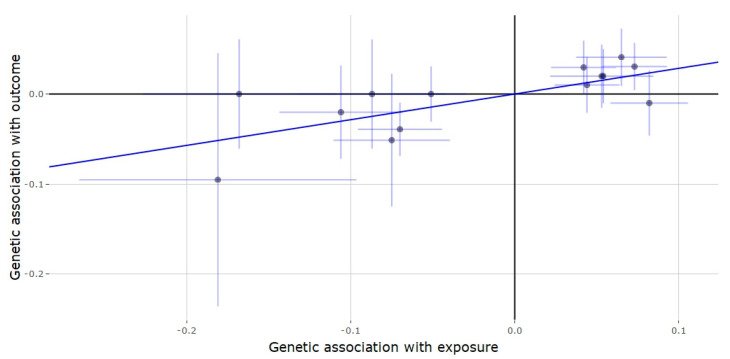
Genetic association of rheumatoid arthritis (RA) against genetic association of testosterone (*SHBG* locus). The slope (dark blue line) represents the causal association of testosterone with RA (generated via inverse variance weighted Mendelian randomization), while the points on the plot and the lights around them (light blue lines) represent the SNP and its confidence intervals.

**Figure 6:**
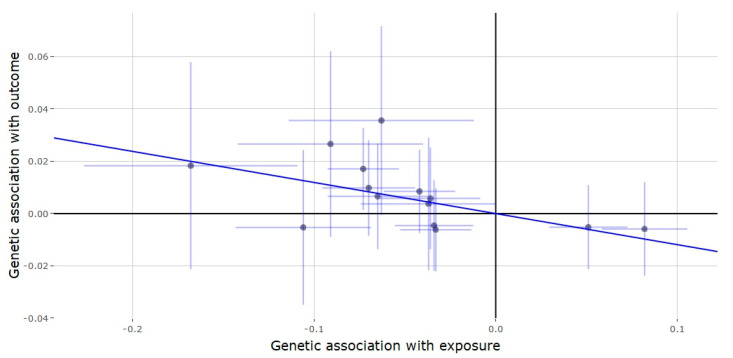
Genetic association of type II diabetes (T2D) against genetic association of testosterone (*SHBG* locus). The slope (dark blue line) represents the causal association of testosterone with T2D (generated via inverse variance weighted Mendelian randomization), while the points on the plot and the lights around them (light blue lines) represent the SNP and its confidence intervals.

Results of the Mendelian randomization analysis of predictors of testosterone from the SHBG region (Table 3) showed that genetically predicted testosterone was positively associated with depression and RA, while negatively associated with T2D. Each SD increase in genetically predicted testosterone was associated with a 1.02-fold increased risk of depression (estimate = 0.021, 95% CI = 0.008, 0.031, *p* = 0.001) (Figure 4). During the sensitivity analysis, the weighted median method showed similar results to the IVW analysis (estimate = 0.016, *p* = 0.040), while there was no evidence for pleiotropy (MR-Egger intercept = 0.002, *p* = 0.092). For RA, each SD increase in testosterone resulted in a 1.33-fold increase in the risk of RA (estimate = 0.285, 95% CI = 0.141, 0.429, *p* < 0.001) (Figure 5), which was similar to the estimates from the weighted median method sensitivity analysis (estimate = 0.368, *p* < 0.001) without any evidence of horizontal pleiotropy (MR-Egger intercept = 0.022, 95% CI = −0.006, 0.049, *p* = 0.120). Each SD increase in testosterone correlated to a 0.887-fold increase in the risk of T2D (estimate = −0.119, 95% CI = −0.203, −0.035, *p* = 0.005) (Figure 6), which was mirrored by the weighted median method analysis (estimate = −0.107, 95% CI = −0.219, −0.005, *p* = 0.062), without any horizontal pleiotropy detected (MR-Egger intercept estimate = 0.003, *p* = 0.219). Genetic association of genetically predicted testosterone with SCZ, BP, T2D and AD were null; however, the direction of effect for T2D was in agreement with that of the significant effect found in the *JMJD1C* analysis. 

## 4. Discussion

Serum testosterone levels naturally decrease over the course of a man’s life or could be reduced as a result of hypogonadism. In this study, we aimed to explore the possible effects of this decrease on common diseases by Mendelian randomization, using genetically predicted testosterone levels derived from two gene regions significantly associated with serum testosterone levels. Mendelian randomization analyses were performed separately on the two instruments; results from the JMJD1C and SHBG loci showed significant associations with three outcomes each. We investigated a total of seven diseases, namely, T2D, SCZ, BP, AD, gout, RA and depression, of which four outcomes displayed significant associations with genetically predicted testosterone levels. Analyses of both loci supported significant associations of genetically predicted testosterone with RA and T2D, while also displaying the same direction of effect.

The results of our study show that an increase in serum testosterone has a protective effect on the risk of developing T2D, with both loci displaying significant results with consistent direction of effect. Existing literature has extensively studied the relationship between serum testosterone and T2D, and found testosterone can directly regulate glucose metabolism [41]; additionally, low levels of serum testosterone were also associated with reduced insulin sensitivity [42]. Testosterone plays an important role in glucose metabolism as it has been shown to increase expression of insulin receptors as well modify the glucose transporter (GLUT4) [43]. Testosterone could also regulate glucose homeostasis and glucose uptake via androgen receptor (AR)-dependent pathways in brain, adipose and pancreatic tissues, as well as AR-independent pathways, for example, by activating the LKB1/AMPK signaling pathway in adipocytes [44]. Impairment of the AMPK pathway has been observed in T2D patients, activation of which results in improved uptake of glucose [45]. Interestingly, activation of the LKB1/AMPK pathway has been shown to result in greater amyloid-β generation [46]. A recent meta-analysis investigating the relationship between low serum testosterone and risk of AD concluded that low serum testosterone was significantly associated with increased risk of AD [33]; however, this may be a characteristic of disease progression rather than a causal relationship. Alternatively, LKB1/AMPK activation via testosterone earlier in life could potentially cause the build-up of amyloid-β plaques and cause AD. T2D and AD share a common pathology to some extent, as both diseases display insulin signaling defects and resistance [47], while testosterone may play a crucial role in the pathogenesis of both these disorders. Additionally, hyperglycaemia, which could result, has also been shown to reduce serum testosterone levels [48,49], which indicates that these relationships are not as straight forward as one may think, as elevated glucose levels result in an increase in adipose tissue, which in turn result in a reduction in testosterone production [50]. 

Historically low levels of serum testosterone have been regularly associated with RA [51,52], and the hypothalamic–pituitary–adrenal axis has been shown to be affected by RA [53], potentially leading to patients affected by RA to display aberrant testosterone levels [54]. In our analysis, we show that increased serum testosterone increases the risk of developing RA, and a recent study investigating the future risk of developing RA in men concluded that low serum testosterone is associated with a rheumatoid factor (RF) negative prediction [29], suggesting the low levels of testosterone may provide protection against development of RA. 

Hypogonadism has been linked to depression in males [55,56], while testosterone has potential antidepressive properties [57]; our results surprisingly suggest that increased testosterone may actually slightly increase the risk of depression.

## 5. Conclusions

In conclusion, our Mendelian randomization study assessed the effect of testosterone on the risk of several common diseases. We report that testosterone has a protective effect on the risk of T2D and gout, while having adverse effects on depression and RA. There were, however, a few limitations of our study, first and foremost being that the genetic instruments used in our Mendelian randomization were sourced from variant testosterone associations measured in men; therefore, our findings are largely relevant to common diseases in men. Another limitation of our study was the use of variants in the SHBG region to genetically predict testosterone. SHBG can bind to free testosterone, thereby affecting the concentrations of testosterone in the blood; though SHBG can be linked to testosterone levels mechanistically, it is difficult to distinguish between the effects of testosterone from that of SHBG.

Our findings, along with previous Mendelian randomization studies on the effect of testosterone on obesity and adverse cardiac events, highlight the importance of testosterone in overall health and well-being, particularly in males. The results of our study show that further research into the effects of testosterone is required, especially in light of the increasing utilization of testosterone therapy.

## Figures and Tables

**Table 1:** Detailed composition of the genetic variants used in the Mendelian randomization analysis.

***JMJD1C*** **Locus**						
**SNP**	**Position**	**Beta**	**Beta (se)**	***p*** **-Value**	**EA**	**OA**
rs10822184	65007159	−0.058	0.01	1.12 × 10^−8^	T	C
***SHBG*** **Locus**						
**SNP**	**Position**	**Beta**	**Beta (se)**	***p*** **-Value**	**EA**	**OA**
rs727428	7478517	−0.073	0.01	1.26 × 10^−12^	T	C
rs1799941	7474148	0.082	0.012	1.39 × 10^−12^	A	G
rs17806566	7292887	−0.168	0.03	2.61 × 10^−8^	C	T
rs9913778	7474626	−0.106	0.019	3.05 × 10^−8^	T	C
rs9900162	7387788	−0.07	0.013	6.13 × 10^−8^	G	A
rs35894069	7335900	0.054	0.011	5.74 × 10^−7^	A	G
rs9908275	7367048	−0.065	0.014	1.59 × 10^−6^	T	C
rs4511593	7396260	0.051	0.011	1.73 × 10^−6^	C	T
rs55784804	7477185	−0.075	0.018	2.25 × 10^−5^	T	G
rs3853818	7287026	−0.044	0.01	2.36 × 10^−5^	T	C
rs12944954	7425855	−0.181	0.043	2.88 × 10^−5^	G	A
rs55894190	7323962	−0.042	0.01	6.90 × 10^−5^	C	T
rs8069501	7335692	−0.087	0.023	1.52 × 10^−4^	G	A
rs858517	7474996	−0.091	0.026	4.44 × 10^−4^	C	T
rs2955611	7490299	−0.053	0.016	9.08 × 10^−4^	C	A
rs12942088	7423503	−0.033	0.01	1.59 × 10^−3^	C	T
rs2302762	7299585	−0.034	0.011	3.06 × 10^−3^	T	C
rs12936934	7441490	−0.036	0.014	9.74 × 10^−3^	T	C
rs4968211	7399786	−0.063	0.026	1.4 × 10^−2^	A	G
rs4796305	7276779	−0.037	0.019	4.6 × 10^−2^	G	T

**Table 2:** Mendelian Randomization (MR) using variants from the *JMJD1C* Locus—Inverse Variance Weighted (IVW) Method.

Outcome	OR	Estimate (se)	*p*-Value
Alzheimer’s Disorder	0.875	−0.133 (0.145)	0.359
Bipolar Disorder	1.402	0.338 (0.234)	0.149
Schizophrenia	1.132	0.124 (0.182)	0.496
Depression	0.984	−0.016 (0.020)	0.437
Rheumatoid Arthritis	1.690	0.525 (0.226)	0.020
Gout	0.469	−0.757 (0.221)	0.001
Type 2 Diabetes	0.769	−0.262 (0.133)	0.048

**Table 3:** Mendelian Randomization (MR) using variants from the *SHBG* Locus—Inverse Variance Weighted (IVW) Method.

Outcome	OR	Beta (se)	*p*-Value	MR-Egger Intercept Estimate (*p*-Value)
Alzheimer’s Disorder	0.998	−0.002 (0.029)	0.935	0.013 (0.015)
Bipolar Disorder	0.991	−0.009 (0.085)	0.920	0.007 (0.664)
Schizophrenia	1.038	0.038 (0.053)	0.176	−0.004 (0.672)
Depression	1.02	0.020 (0.006)	0.001	0.002 (0.092)
Rheumatoid Arthritis	1.329	0.285 (0.060)	<0.001	0.022 (0.120)
Gout	0.971	−0.029 (0.064)	0.649	0.005 (0.683)
Type 2 Diabetes	0.887	−0.119 (0.030)	0.003	−0.027 (0.607)

## Supplementary Materials

The following are available online at https://www.mdpi.com/2073-4425/11/7/721/s1. Figure S1: LD matrix—depression, *SHBG* instrument locus; Figure S2: LD matrix—RA instrument, *SHBG* locus; Figure S3: LD matrix—T2D instrument, *SHBG* locus; Table S1: Depression, *SHBG* instrument locus; Table S2: RA instrument, *SHBG* locus; Table S3: T2D instrument, *SHBG* locus.

## References

[1] Mooradian A.D., Morley J.E., Korenman S.G. (1987). Biological Actions of Androgens. Endocr. Rev..

[2] Laughlin G.A., Barrett-Connor E., Bergstrom J. (2008). Low Serum Testosterone and Mortality in Older Men. J. Clin. Endocrinol. Metab..

[3] Shores M.M., Matsumoto A.M., Sloan K.L., Kivlahan D.R. (2006). Low Serum Testosterone and Mortality in Male Veterans. Arch. Intern. Med..

[4] Bhasin S., Cunningham G.R., Hayes F.J., Matsumoto A.M., Snyder P.J., Swerdloff R.S., Montori V.M. (2010). Testosterone Therapy in Men with Androgen Deficiency Syndromes: An Endocrine Society Clinical Practice Guideline. J. Clin. Endocrinol. Metab..

[5] Feldman H.A., Longcope C., Derby C.A., Johannes C.B., Araujo A.B., Cobiello A.D., Bremner W.J., McKinlay J.B. (2002). Age trends in the level of serum testosterone and other hormones in middle-aged men: Longitudinal results from the Massachusetts male aging study. J. Clin. Endocrinol. Metab..

[6] Harman S.M., Meteer E.J., Tobin J.D., Pearson J., Blackman M.R. (2001). Longitudinal effects of aging on serum total and free testosterone levels in healthy men. Baltimore Longitudinal Study of Aging. J. Clin. Endocrinol. Metab..

[7] Wu F., Tajar A., Pye S., Silman A.J., Finn J.D., O’Neill T.W., Bartfai G., Casanueva F., Forti G., Giwercman A. (2008). Hypothalamic-Pituitary-Testicular Axis Disruptions in Older Men Are Differentially Linked to Age and Modifiable Risk Factors: The European Male Aging Study. J. Clin. Endocrinol. Metab..

[8] Mulligan T., Frick M.F., Zuraw Q.C., Stemhagen A., McWhirter C. (2008). Prevalence of hypogonadism in males aged at least 45 years: The HIM study. Int. J. Clin. Pract..

[9] Layton J.B., Li N., Meier C.R., Sharpless J.L., Stürmer T., Jick S., Brookhart M.A. (2014). Testosterone lab testing and initiation in the United Kingdom and the United States, 2000 to 2011. J. Clin. Endocrinol. Metab..

[10] Elliott J., E Kelly S., Millar A.C., Peterson J., Chen L., Johnston A., Kotb A., Skidmore B., Bai Z., Mamdani M. (2017). Testosterone therapy in hypogonadal men: A systematic review and network meta-analysis. BMJ Open.

[11] Canguven O., Talib R.A., El Ansari W., Yassin D., Salman M., Al-Ansari A. (2017). Testosterone therapy has positive effects on anthropometric measures, metabolic syndrome components (obesity, lipid profile, Diabetes Mellitus control), blood indices, liver enzymes, and prostate health indicators in elderly hypogonadal men. Andrologia.

[12] Gagliano-Jucá T., Basaria S. (2019). Testosterone replacement therapy and cardiovascular risk. Nat. Rev. Cardiol..

[13] Finkle W.D., Greenland S., Ridgeway G., Adams J.L., Frasco M., Cook M.B., Fraumeni J.F., Hoover R.N. (2014). Increased Risk of Non-Fatal Myocardial Infarction Following Testosterone Therapy Prescription in Men. PLoS ONE.

[14] Vigen R., O’Donnell C.I., Barón A.E., Grunwald G.K., Maddox T.M., Bradley S.M., Barqawi A., Woning G., Wierman M.E., Plomondon M.E. (2013). Association of Testosterone Therapy with Mortality, Myocardial Infarction, and Stroke in Men With Low Testosterone Levels. JAMA.

[15] Carré J.M., Archer J. (2018). Testosterone and human behavior: The role of individual and contextual variables. Curr. Opin. Psychol..

[16] Dreher J.-C., Dunne S., Pazderska A., Frodl T., Nolan J.J., O’Doherty J.P. (2016). Testosterone causes both prosocial and antisocial status-enhancing behaviors in human males. Proc. Natl. Acad. Sci. USA.

[17] Hua J.T., Hildreth K.L., Pelak V.S. (2016). Effects of Testosterone Therapy on Cognitive Function in Aging. Cogn. Behav. Neurol..

[18] Corona G., Mannucci E., Ricca V., Lotti F., Boddi V., Bandini E., Balercia G., Forti G., Maggi M. (2009). The age-related decline of testosterone is associated with different specific symptoms and signs in patients with sexual dysfunction. Int. J. Androl..

[19] Gapstur S.M., Gann P.H., Kopp P., Colangelo L., Longcope C., Liu K. (2002). Serum androgen concentrations in young men: A longitudinal analysis of associations with age, obesity, and race. The CARDIA male hormone study. Cancer Epidemiol. Biomark. Prev..

[20] Jensen M.B., Andersson A.-M., Jørgensen N., Andersen A.-G., Carlsen E., Petersen J.H., Skakkebæk N.E. (2004). Body mass index in relation to semen quality and reproductive hormonesamong 1558 Danish men. Fertil. Steril..

[21] Svartberg J., Von Mühlen D., Sundsfjord J., Jorde R. (2004). Waist circumference and testosterone levels in community dwelling men. The Tromsø study. Eur. J. Epidemiol..

[22] Zhao J.V., Jiang C., Lam T.H., Liu B., Cheng K., Xu L., Yeung S.L.A., Zhang W., Leung G., Schooling M. (2013). Genetically predicted testosterone and cardiovascular risk factors in men: A Mendelian randomization analysis in the Guangzhou Biobank Cohort Study. Int. J. Epidemiol..

[23] Zhao J.V., Jiang C., Lam T.H., Liu B., Cheng K., Xu L., Yeung S.L.A., Zhang W., Leung G., Schooling M. (2015). Genetically Predicted Testosterone and Systemic Inflammation in Men: A Separate-Sample Mendelian Randomization Analysis in Older Chinese Men. PLoS ONE.

[24] Zhao J.V., Lam T.H., Jiang C., Cherny S.S., Liu B., Cheng K.K., Zhang W., Leung G., Schooling M. (2016). A Mendelian randomization study of testosterone and cognition in men. Sci. Rep..

[25] Ruth K.S., Day F.R., Tyrrell J., Thompson D.J., Wood A.R., Mahajan A., Beaumont R.N., Wittemans L., Martin S., Busch A.S. (2020). Using human genetics to understand the disease impacts of testosterone in men and women. Nat. Med..

[26] Schooling M., Luo S., Yeung S.L.A., Thompson D.J., Karthikeyan S., Bolton T.R., Mason A.M., Ingelsson E., Burgess S. (2018). Genetic predictors of testosterone and their associations with cardiovascular disease and risk factors: A Mendelian randomization investigation. Int. J. Cardiol..

[27] Paruk I.M., Pirie F.J., Nkwanyana N.M., Motala A.A. (2019). Prevalence of low serum testosterone levels among men with type 2 diabetes mellitus attending two outpatient diabetes clinics in KwaZulu-Natal Province, South Africa. S. Afr. Med. J..

[28] Pui K., Waddell C., Dalbeth N. (2008). Early onset of hyperuricaemia and gout following treatment for female to male gender reassignment. Rheumatology.

[29] Pikwer M., Giwercman A., Bergström U., Nilsson J., Jacobsson L.T.H., Turesson C. (2013). Association between testosterone levels and risk of future rheumatoid arthritis in men: A population-based case–control study. Ann. Rheum. Dis..

[30] Amiaz R., Seidman S.N. (2008). Testosterone and depression in men. Curr. Opin. Endocrinol. Diabetes Obes..

[31] Nead K.T. (2019). Androgens and depression. Curr. Opin. Endocrinol. Diabetes Obes..

[32] Wooderson S.C., Gallagher P., Watson S., Young A.H. (2015). An exploration of testosterone levels in patients with bipolar disorder. BJPsych Open.

[33] Lv W., Du N., Liu Y., Fan X., Wang Y., Jia X., Hou X., Wang B. (2015). Low Testosterone Level and Risk of Alzheimer’s Disease in the Elderly Men: A Systematic Review and Meta-Analysis. Mol. Neurobiol..

[34] Jin G., Sun J., Kim S.-T., Feng J., Wang Z., Tao S., Chen Z., Purcell L., Smith S., Isaacs W.B. (2012). Genome-wide association study identifies a new locus JMJD1C at 10q21 that may influence serum androgen levels in men. Hum. Mol. Genet..

[35] Xue A., Wu Y., Zhu Z., Zhang F., Kemper K.E., Zheng Z., Yengo L., Lloyd-Jones L.R., Sidorenko J., Wu Y. (2018). Genome-wide association analyses identify 143 risk variants and putative regulatory mechanisms for type 2 diabetes. Nat. Commun..

[36] Tin A., Marten J., Halperin Kuhns V.L., Li Y., Wuttke M., Kirsten H., Sieber K.B., Qiu C., Gorski M., Yu Z. (2019). Target genes, variants, tissues and transcriptional pathways influencing human serum urate levels. Nat. Genet..

[37] Okada Y., Wu D., Trynka G., Raj T., Terao C., Ikari K., Kochi Y., Ohmura K., Suzuki A., Yoshida S. (2013). Genetics of rheumatoid arthritis contributes to biology and drug discovery. Nature.

[38] Howard D.M., Adams M.J., Shirali M., Clarke T.-K., Marioni R.E., Davies G., Coleman J.R.I., Alloza C., Shen X., Barbu M.C. (2018). Genome-wide association study of depression phenotypes in UK Biobank identifies variants in excitatory synaptic pathways. Nat. Commun..

[39] Schizophrenia Working Group of the Psychiatric Genomics Consortium (2014). Biological insights from 108 schizophrenia-associated genetic loci. Nature.

[40] Burgess S., Thompson S.G. (2017). Interpreting findings from Mendelian randomization using the MR-Egger method. Eur. J. Epidemiol..

[41] Kim C., Halter J.B. (2014). Endogenous sex hormones, metabolic syndrome, and diabetes in men and women. Curr. Cardiol. Rep..

[42] Yialamas M.A., Dwyer A.A., Hanley E., Lee H., Pitteloud N., Hayes F.J. (2007). Acute Sex Steroid Withdrawal Reduces Insulin Sensitivity in Healthy Men with Idiopathic Hypogonadotropic Hypogonadism. J. Clin. Endocrinol. Metab..

[43] Rao P.M., Kelly D., Jones T.H. (2013). Testosterone and insulin resistance in the metabolic syndrome and T2DM in men. Nat. Rev. Endocrinol..

[44] Mitsuhashi K., Senmaru T., Fukuda T., Yamazaki M., Shinomiya K., Ueno M., Kinoshita S., Kitawaki J., Katsuyama M., Tsujikawa M. (2016). Testosterone stimulates glucose uptake and GLUT4 translocation through LKB1/AMPK signaling in 3T3-L1 adipocytes. Endocrine.

[45] Coughlan K.A., Valentine R.J., Ruderman N.B., Saha A.K. (2014). AMPK activation: A therapeutic target for type 2 diabetes?. Diabetes Metab. Syndr. Obes. Targets Ther..

[46] Cai Z., Yan L.-J., Li K., Quazi S.H., Zhao B. (2012). Roles of AMP-activated Protein Kinase in Alzheimer’s Disease. Neuromol. Med..

[47] Gabbouj S., Ryhänen S., Marttinen M., Wittrahm R., Takalo M., Kemppainen S., Martiskainen H., Tanila H., Haapasalo A., Hiltunen M. (2019). Altered Insulin Signaling in Alzheimer’s Disease Brain—Special Emphasis on PI3K-Akt Pathway. Front. Mol. Neurosci..

[48] Caronia L.M., Dwyer A.A., Hayden U., Amati F., Pitteloud N., Hayes F.J. (2013). Abrupt decrease in serum testosterone levels after an oral glucose load in men: Implications for screening for hypogonadism. Clin. Endocrinol..

[49] Seidell J.C., Björntorp P., Sjöström L., Kvist H., Sannerstedt R. (1990). Visceral fat accumulation in men is positively associated with insulin, glucose, and C-peptide levels, but negatively with testosterone levels. Metabolism.

[50] Grossmann M. (2013). Testosterone and glucose metabolism in men: Current concepts and controversies. J. Endocrinol..

[51] Martens H.F., Sheets P.K., Tenover J.S., E Dugowson C., Bremner W.J., Starkebaum G. (1994). Decreased testosterone levels in men with rheumatoid arthritis: Effect of low dose prednisone therapy. J. Rheumatol..

[52] Spector T.D., A Perry L., Tubb G., Silman A.J., Huskisson E.C. (1988). Low free testosterone levels in rheumatoid arthritis. Ann. Rheum. Dis..

[53] Geenen R., Van Middendorp H., Bijlsma J.W. (2006). The Impact of Stressors on Health Status and Hypothalamic-Pituitary-Adrenal Axis and Autonomic Nervous System Responsiveness in Rheumatoid Arthritis. Ann. N. Y. Acad. Sci..

[54] Lashkari M., Noori A., Oveisi S., Kheirkhah M. (2018). Association of serum testosterone and dehydroepiandrosterone sulfate with rheumatoid arthritis: A case control study. Electron. Physician.

[55] Seidman S.N., Walsh B.T. (1999). Testosterone and depression in aging men. Am. J. Geriatr. Psychiatry.

[56] McHenry J., Carrier N., Hull E., Kabbaj M. (2013). Sex differences in anxiety and depression: Role of testosterone. Front. Neuroendocrinol..

[57] Zarrouf F.A., Artz S., Griffith J., Sirbu C., Kommor M. (2009). Testosterone and Depression. Am. J. Geriatr. Psychiatry.

