# Supplementary figures and images for: The Potential Effect of Aberrant Testosterone Levels on Common Diseases: A Mendelian Randomization Study
Source: Genes (Basel). 2020 Jun 29;11(7):721. doi: 10.3390/genes11070721 (PMC7397292; doi:10.3390/genes11070721)

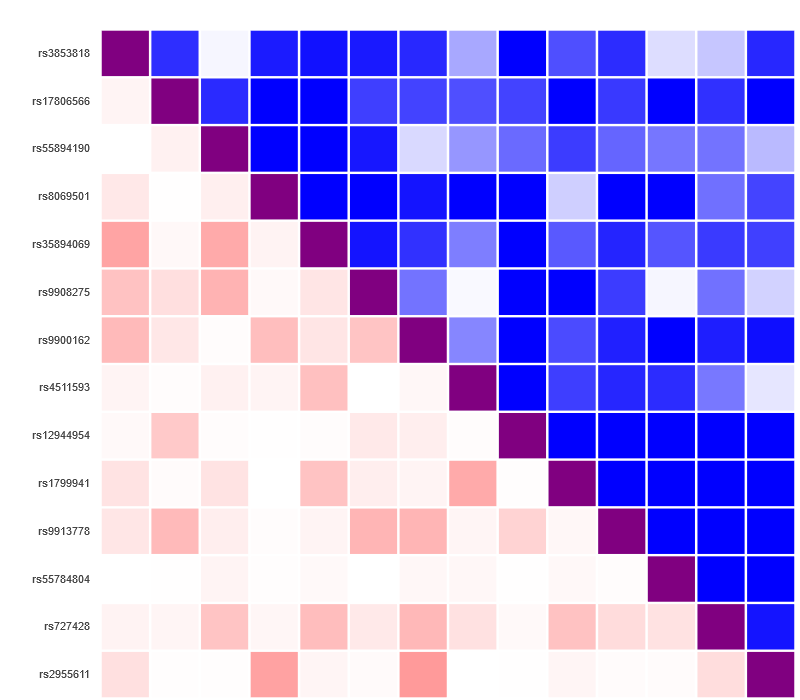

Supplement: Supplementary file 1 [file genes-11-00721-s001.zip › LD matrix Depression SHBG instrument Locus.png]

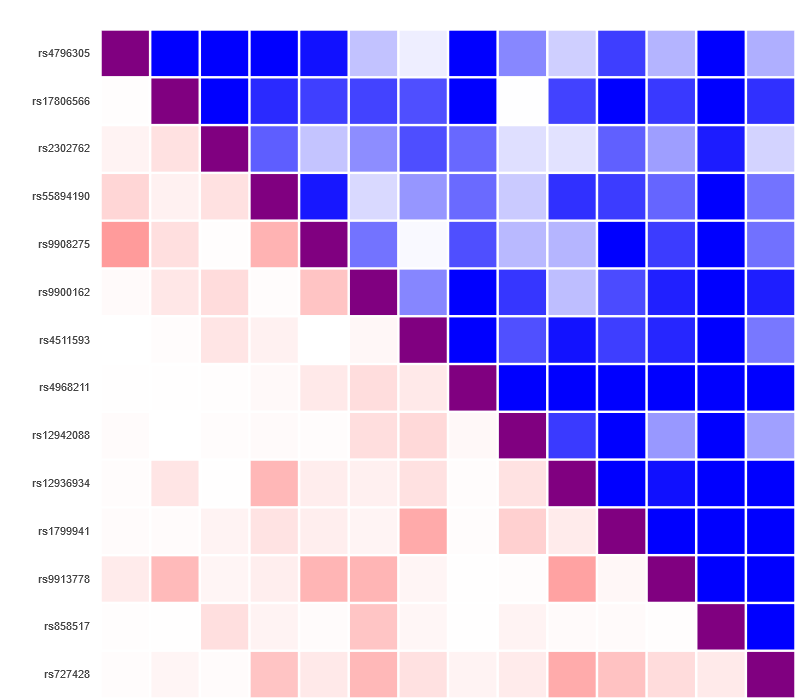

Supplement: Supplementary file 1 [file genes-11-00721-s001.zip › LD-matrix T2D instrument SHBG Locus.png]
